# Assessment of Dietary Choline Intake, Contributing Food Items, and Associations with One-Carbon and Lipid Metabolites in Middle-Aged and Elderly Adults: The Hordaland Health Study

**DOI:** 10.1093/jn/nxab367

**Published:** 2021-10-13

**Authors:** Anthea Van Parys, Maria Sandvik Brække, Therese Karlsson, Kathrine J Vinknes, Grethe S Tell, Teresa R Haugsgjerd, Per Magne Ueland, Jannike Øyen, Jutta Dierkes, Ottar Nygård, Vegard Lysne

**Affiliations:** Centre for Nutrition, Department of Clinical Science, University of Bergen, Bergen, Norway; Centre for Nutrition, Department of Clinical Science, University of Bergen, Bergen, Norway; Department of Internal Medicine and Clinical Nutrition, Sahlgrenska Academy, University of Gothenburg, Gothenburg, Sweden; Department of Nutrition, Institute of Basic Medical Sciences, Faculty of Medicine, University of Oslo, Oslo, Norway; Department of Global Public Health and Primary Care, University of Bergen, Bergen, Norway; Department of Global Public Health and Primary Care, University of Bergen, Bergen, Norway; Bevital, Bergen, Norway; Institute of Marine Research, Bergen, Norway; Mohn Nutrition Research Laboratory, University of Bergen, Bergen, Norway; Centre for Nutrition, Department of Clinical Medicine, University of Bergen, Bergen, Norway; Department of Laboratory Medicine and Pathology, Haukeland University Hospital, Bergen, Norway; Centre for Nutrition, Department of Clinical Science, University of Bergen, Bergen, Norway; Mohn Nutrition Research Laboratory, University of Bergen, Bergen, Norway; Department of Heart Disease, Haukeland University Hospital, Bergen, Norway; Centre for Nutrition, Department of Clinical Science, University of Bergen, Bergen, Norway; Mohn Nutrition Research Laboratory, University of Bergen, Bergen, Norway; Department of Heart Disease, Haukeland University Hospital, Bergen, Norway

**Keywords:** choline, dietary intake, one-carbon metabolism, lipid metabolism, phosphatidylcholine

## Abstract

**Background:**

Choline is an essential nutrient for humans and is involved in various physiologic functions. Through its metabolite betaine, it is closely connected to the one-carbon metabolism, and the fat-soluble choline form phosphatidylcholine is essential for VLDL synthesis and secretion in the liver connecting choline to the lipid metabolism. Dietary recommendations for choline are not available in the Nordic countries primarily due to data scarcity.

**Objectives:**

The aim of this study was to investigate the dietary intake of total choline and individual choline forms, dietary sources, and the association of total choline intake with circulating one-carbon metabolites and lipids.

**Methods:**

We included 5746 participants in the Hordaland Health Study, a survey including community-dwelling adults born in 1925–1927 (mean age 72 y, 55% women) and 1950–1951 (mean age 48 y, 57% women). Dietary data were obtained using a 169-item FFQ, and choline content was calculated using the USDA Database for Choline Content of Common Foods, release 2. Metabolites of the one-carbon and lipid metabolism were measured in a nonfasting blood sample obtained at baseline, and the association with total choline intake was assessed using polynomial splines.

**Results:**

The geometric mean (95% prediction interval) energy-adjusted total choline intake was 260 (170, 389) mg/d, with phosphatidylcholine being the main form (44%). The major food items providing dietary choline were eggs, low-fat milk, potatoes, and leafy vegetables. Dietary total choline was inversely associated with circulating concentrations of total homocysteine, glycine, and serine and positively associated with choline, methionine, cystathionine, cysteine, trimethyllysine, trimethylamine-N-oxide, and dimethylglycine. A weak association was observed between choline intake and serum lipids.

**Conclusions:**

Phosphatidylcholine was the most consumed choline form in community-dwelling adults in Norway. Our findings suggest that choline intake is associated with the concentration of most metabolites involved in the one-carbon and lipid metabolism.

## Introduction

Since 1998, choline has been recognized as an essential nutrient, as de novo synthesis was proven to be insufficient (1). It is involved in liver, muscle, and brain functioning and plays a role in diverse processes such as cellular signaling, hepatic lipid metabolism, and methylation-dependent biosynthesis of molecules, including epigenetic regulation and gene expression (2, 3). In addition, phosphorylated choline compounds are elementary structural phospholipids in most cell membranes (4). Phosphatidylcholine (PC) and sphingomyelin are both lipid-soluble choline forms, whereas glycerophosphocholine, phosphocholine, and free choline are water soluble. All forms are found in the diet, with PC being the most abundant. Overall, total choline content per unit of weight is higher in animal food sources compared with plant-based sources, which makes eggs, meat, fish, chicken, and dairy good choline sources (5). Nonetheless, plant-based foods such as leafy vegetables, potatoes, grain products, nuts and seeds, and most legumes are also good choline sources, primarily containing water-soluble choline forms.

So far, an adequate intake (AI) for total dietary choline has been set by the National Academy of Medicine (NAM) in the United States (550 mg/d for men and 425 mg/d for women) (1) and by the European Food Safety Authority (EFSA) (400 mg/d for adults) (6). Due to limited scientific evidence, neither of these agencies have published an estimated average requirement. Also, no recommendations have been published as per now for the Nordic countries (7). Choline intake has been investigated in a range of countries worldwide, and even though it has varied largely between investigated countries, the estimated dietary choline intake has mainly been below the recommendations from both NAM and EFSA in most of the studied populations (8–15). So far, the intake of individual choline forms has been investigated in only a few countries (5). Little is known about the consumption and dietary sources of choline and the different forms globally, including in Norway. Because inadequate intake could possibly lead to adverse health effects such as muscle or liver damage (16, 17), choline intake and dietary sources should be investigated to allow for establishment of dietary recommendations.

Choline is closely connected to the one-carbon metabolism, a set of biochemical reactions in which one-carbon groups are being transferred between compounds through its metabolite betaine. Disturbances in metabolites involved in the one-carbon metabolism have been linked to the development of several chronic diseases (18). Other dietary-derived compounds such as folate, betaine, riboflavin, vitamin B-6, cobalamin, and methionine can also generate methyl groups, and their metabolisms are therefore closely related to that of choline (18, 19). For example, choline deficiency may lead to increased de novo choline synthesis via sequential methylation of phosphatidylethanolamine by S-adenosylmethionine, generated from methionine (18). Sex also alters one's choline requirement as estrogen promotes de novo synthesis. Therefore, men and postmenopausal women have a higher choline need compared with premenopausal women (20). Several other factors such as pregnancy, lactation, and gene polymorphisms can also affect one's choline requirement (21). The one-carbon metabolism is complex and characterized by many feedback mechanisms. Even though the association with dietary choline and some one-carbon metabolites has been investigated [e.g., total homocysteine (22) and plasma choline (16)], it remains unclear for several others.

Choline is also involved in the lipid metabolism mainly through PC, which is required for solubilization of bile salts for secretion and, most important, for packaging and export of triglycerides (TGs) from the liver, as a structural part of VLDL (3, 4). Despite the clear association between choline and lipid metabolism, little research has been conducted regarding the relation between choline intake and serum lipids in humans.

The aim of this study was to describe the dietary intake of total choline and the individual choline forms in a community-based population. In addition, the contributions of different dietary choline sources to total choline and individual choline forms were explored. Finally, associations between total dietary choline intake and circulating concentrations of both one-carbon and lipid metabolites were investigated.

## Methods

### Study population

This study uses data from the community-based Hordaland Health Study (HUSK) (https://husk.w.uib.no/) conducted in western Norway. The recruitment of this cohort in 1997–1999 was based on a previous cohort from 1992–1993 (The Hordaland Homocysteine Study) in which all individuals living in Hordaland county (currently part of Vestland county) born in 1925–1927 or 1950–1952 were invited. In 1997–1999, all living participants born in 1925–1927 or 1950–1951 and residing in the city of Bergen or the neighboring municipalities were reinvited. The main purpose of the HUSK surveys is to gather information for prevention of future disease through *1*) prevention via identification of possible modifiable risk factors for disease and *2*) research via mapping of occurrence to be able to identify the extent of illness, identify causal factors, and be able to better predict future needs for health services. Participants underwent a brief health examination and provided a nonfasting blood sample at baseline. Data from 7016 HUSK participants were available for this study, of whom 6094 completed the FFQ. We excluded 30 participants with missing plasma choline values. Furthermore, participants with extreme energy intakes (<3300 kJ or >17,500 kJ for men and <3000 kJ or >15,000 kJ for women) were excluded (*n* = 198). Finally, we excluded participants with self-reported alcohol intake >10 energy percentage (E%)  (*n* = 120). This left 5746 participants eligible for analyses. Excluded participants comprised 306 elderly women, 219 elderly men, 350 middle-aged women, and 395 middle-aged men. All study participants provided written informed consent, and the study protocol was approved by the regional ethics committee for Medical Research Ethics.

### Health examination and analytic procedures

Information regarding lifestyle, health behaviors, and medical history was obtained through self-administered questionnaires. Smoking was defined based on self-reported smoking habits and serum cotinine concentrations >85 nmol/L. Participants were classified as having diabetes according to self-report. Hypertension was considered present if the participant reported use of medication for hypertension.

Study procedures for HUSK have been described in detail previously (23). Briefly, participants underwent a brief physical examination, which included measurements of height and weight, and a venous, nonfasting blood sample was taken. Blood samples were collected in evacuated tubes containing EDTA, chilled (at 4–5°C) within 15–30 min, and then centrifuged for 10 min, 4000 × *g* at 10°C within 1–3 h and stored at –80°C until analysis. Metabolites related to one-carbon metabolism and cotinine, as a biomarker of nicotine exposure, were analyzed in 5746 samples at Bevital AS. Betaine, choline, dimethylglycine (DMG), trimethylamine-N-oxide (TMAO), trimethyllysine (TML), and cotinine were measured using LC-MS/MS (24, 25), whereas GC-MS/MS was used for cystathionine, cysteine, glycine, methionine, serine, and total homocysteine analyses (26). Serum samples of total cholesterol (TC), HDL cholesterol, and TGs were analyzed within 7 d at the Department of Clinical Chemistry, Oslo University Hospital, Ullevål, with reagents from Boehringer Mannheim (Roche) as adapted to a Hitachi 911 analyzer. Cholesterol and TGs were measured by enzymatic methods. HDL cholesterol was measured by a direct, enzymatic inhibition method. LDL cholesterol concentration was calculated using the Friedewald equation: LDL cholesterol (mmol/L) = TC (mmol/L) – HDL cholesterol (mmol/L) – TG (mmol/L) / 2.2 (27). Serum LDL was not calculated for participants with serum TG >4.5 mmol/L as the Friedewald equation is not applicable in this case (*n* = 137). Non-HDL-LDL cholesterol was used as a surrogate marker for cholesterol in chylomicrons and calculated using the following formula: non-HDL-LDL cholesterol (mmol/L) = TC (mmol/L) – HDL cholesterol (mmol/L) – LDL cholesterol (mmol/L). The number of participants with available results per metabolite is shown in **Table 1**.

**TABLE 1:** Number of participants with available results per metabolite

		Elderly		Middle-aged	
	Total population	Women	Men	Women	Men
*n* (%)	5746	1539 (26.8)	1247 (21.7)	1700 (29.6)	1260 (21.9)
One-carbon metabolites, *n*					
Betaine	5570	1501	1199	1669	1201
Choline	5746	1539	1247	1700	1260
Cystathionine	5653	1525	1221	1683	1224
Cysteine	5653	1525	1221	1683	1224
DMG	5746	1539	1247	1700	1260
Glycine	5653	1525	1221	1683	1224
Methionine	5653	1525	1221	1683	1224
Serine	5653	1525	1221	1683	1224
TMAO	5570	1501	1199	1669	1201
TML	5570	1501	1199	1669	1201
Total homocysteine	5653	1525	1221	1683	1224
Serum lipids, *n*					
Total cholesterol	5746	1539	1247	1700	1260
LDL cholesterol	5609	1509	1220	1671	1209
HDL cholesterol	5746	1539	1247	1700	1260
Non-HDL-LDL cholesterol	5746	1539	1247	1700	1260
Triglycerides	5746	1539	1247	1700	1260

DMG indicates dimethylglycine; HDL, high-density-lipoprotein; LDL, low-density-lipoprotein; TMAO, trimethylamine-N-oxide; TML, trimethyllysine.

### Dietary assessment

Information regarding dietary intake was obtained using a 169-item semiquantitative FFQ, which is a slightly modified version of a previously described FFQ (28). The FFQ was handed to the participants on the day of the health examination, filled out at home, and returned by mail to the HUSK project center. The frequency alternatives ranged from once a month to several times a day. In addition, number of units eaten (e.g., slices, pieces) and household measures (e.g., spoons, cups, glasses) were used to capture habitual diet during the past year. In addition to food items, the FFQ included the 9 most common single- and multivitamin supplements at the time of the study, but no specific questions regarding choline supplementation were included. Daily nutrient intake was calculated using the software system “Kostberegningssystemet” (KBS, version 3.2) developed at the Department of Nutrition, University of Oslo, Norway. The nutrient database used is mainly based on the official Norwegian food composition table (https://www.matvaretabellen.no/).

### Choline composition data

Total choline was defined as the sum of the 5 individual choline forms: free choline, glycerophosphocholine, phosphocholine, PC, and sphingomyelin. As choline composition data are currently unavailable in the Norwegian food composition table, choline content of foods was quantified using the USDA Database for Choline Content of Common Foods, release 2 (29). From the 169-item FFQ, choline content was available for 134 food items. For the remaining items, choline content was estimated using nutritionally equivalent foods. For multicomponent foods (e.g., dishes, ready-made meals), choline content was calculated for each ingredient in the FFQ recipe. The 134 food items were categorized into 10 main food groups (dairy, drinks, eggs, fats, fish, fruit, grain products, meat, vegetables, and sweets and snacks) and subsequently into categories and subcategories based on nutritional similarities. A full overview of food items and categorizations is provided in **Supplemental Table 1**.

### Statistical analyses

Continuous variables are reported as geometric mean [95% prediction interval (PI)] and categorical variables as counts (percentage). The density method was used to adjust dietary variables for self-reported energy intake, and values are reported as E% or g/1000 kcal (30). To adjust dietary choline intake for self-reported energy intake, the residual method was used. The energy-adjusted choline estimate is the residual from the regression model with total energy intake as the independent variable and absolute choline intake as the dependent variable plus the expected nutrient intake for the mean energy intake in the study population (31).

To generate the density plot for total choline intake, we estimated the probability density function using the kernel density estimation (32, 33) implemented via the “geom_density” function of the “ggplot2” package available for R version 1.3.959 (34). The following formula was used to calculate the percent contribution of each (sub)category to both total choline intake and the intake of individual choline forms: [(choline provided by the food (sub)category / total choline from all food (sub)categories)] × 100.

To explore the relation between one-carbon metabolites and dietary choline intake, choline intake was modeled as a polynomial spline in a model with sex as an interaction term and adjusted for age, BMI, and smoking for total choline and the individual choline forms. The same model was used to explore the relation between lipid metabolism metabolites and choline intake. Including statin use, diabetes diagnosis, folate status, or all 3 factors as an interaction term to the used model did not alter the observed associations substantially, and these results are therefore not shown.

Given the exploratory and descriptive nature of this study, the main results are presented visually, and in accordance with the STROBE checklist and statement (35), we chose not to report *P* values.

All statistical analyses were performed using R version 1.3.959 (R Foundation for Statistical Computing), including the packages within the “tidyverse” (“dplyr,” “ggplot2,” “broom,” “plyr,” “ggthemes”) and the “survival,” “splines,” and “interaction” packages.

## Results

### Characteristics of the study participants

The total study population (*n* = 5746) included women and men born in either 1925–1927 or 1950–1951, hereafter referred to as elderly and middle-aged, respectively. There were slightly more women than men among both the elderly and the middle-aged participants (respectively, 26.8% compared with 21.7% and 29.6% compared with 21.9%). Smoking was less common in the elderly participants, whereas diabetes and hypertension prevalence was higher compared with the middle-aged group.

### Dietary intake

The dietary intake of the study population is shown in **Table 2**. As expected, energy intake of men was higher compared with women and also higher in middle-aged compared with elderly participants. The macronutrient intake was similar in all groups. Elderly participants consumed relatively more dairy, eggs, fish, fruit, and vegetables compared with the middle-aged participants, whereas meat intake was higher in middle-aged men and women compared with their older counterparts.

**TABLE 2:** Characteristics of participants in the Hordaland Health Study 1997–1999^1^

		Elderly		Middle-aged	
Characteristic	Total population	Women	Men	Women	Men
*n* (%)	5746	1539 (26.8)	1247 (21.7)	1700 (29.6)	1260 (21.9)
Age, y	58.6 (47.0, 74.0)	72.4 (71.0, 74.0)	72.4 (71.0, 74.0)	48.0 (47.0, 49.0)	48.0 (47.0, 49.0)
BMI, kg/m^2^	25.4 (19.5, 34.6)	25.8 (18.7, 35.5)	25.7 (20.1, 32.5)	24.6 (19.3, 35.1)	25.9 (20.6, 33.9)
Smokers,^2^ *n* (%)	1566 (27.3)	243 (15.8)	247 (19.8)	616 (36.2)	460 (36.5)
Diabetes,^3^ *n* (%)	201 (3.5)	96 (6.2)	80 (6.4)	7 (0.4)	18 (1.4)
Hypertension,^4^ *n* (%)	909 (15.8)	437 (28.4)	342 (27.4)	77 (4.5)	53 (4.2)
Plasma concentration of one-carbon metabolites,^5^ µmol/L					
Betaine (*n* = 5570)	36.1 (19.7, 60.5)	34.1 (20.3, 55.2)	41.4 (26.9, 63.6)	31.2 (17.3, 51.6)	41.2 (27.0, 67.6)
Choline	9.6 (6.2, 15.1)	9.6 (6.0, 15.0)	10.8 (7.1, 16.7)	8.9 (5.8, 13.3)	9.6 (6.3, 14.9)
Cystathionine (*n* = 5653)	0.3 (0.1, 0.9)	0.3 (0.1, 1.1)	0.3 (0.1, 1.2)	0.2 (0.09, 0.5)	0.2 (0.1, 0.8)
Cysteine (*n* = 5653)	286 (232, 353)	306 (258, 368)	304 (256, 363)	264 (222, 318)	277 (236, 329)
DMG	4.5 (2.7, 7.7)	4.3 (2.6, 7.5)	4.7 (2.9, 8.1)	4.2 (2.6, 7.3)	4.7 (3.0, 8.1)
Glycine (*n* = 5653)	251 (158, 457)	268 (159, 492)	230 (158, 350)	270 (151, 474)	230 (162, 350)
Methionine (*n* = 5653)	27.5 (17.6, 49.0)	26.2 (17.3, 49.6)	28.8 (18.1, 52.2)	26.6 (16.9, 46.4)	29.2 (19.3, 49.2)
Serine (*n* = 5653)	114 (77, 171)	114 (76, 169)	109 (75, 160)	121 (79, 183)	111 (78, 161)
TMAO (*n* = 5570)	5.6 (1.6, 49.2)	6.6 (2.0, 60.7)	8.2 (2.1, 72.7)	4.0 (1.3, 28.2)	4.9 (1.4, 42.0)
TML (*n* = 5570)	0.6 (0.3, 1.2)	0.6 (0.4, 1.2)	0.7 (0.4, 1.4)	0.5 (0.3, 1.0)	0.6 (0.4, 1.3)
Total homocysteine (*n* = 5653)	11 (6.6, 21.1)	11.7 (7.2, 21.3)	13.2 (8.3, 23.3)	9.2 (5.8, 16.8)	11.0 (7.2, 19.6)
Serum lipids, mmol/L					
Total cholesterol	5.9 (4.1, 8.3)	6.5 (4.5, 8.9)	5.8 (3.9, 8.0)	5.6 (4.1, 7.7)	5.7 (4.1, 8.0)
LDL cholesterol^6^ (*n* = 5609)	4.5 (2.8, 6.9)	5.0 (3.1, 7.3)	4.5 (2.7, 6.7)	4.1 (2.6, 6.3)	4.5 (2.9, 6.7)
HDL cholesterol	1.3 (0.7, 2.2)	1.4 (0.9, 2.4)	1.2 (0.7, 2.0)	1.4 (0.9, 2.3)	1.1 (0.7, 1.9)
Non-HDL-LDL cholesterol^7^	0.7 (0.3, 0.2)	0.7 (0.3, 1.9)	0.7 (0.3, 2.0)	0.6 (0.2, 1.8)	0.8 (0.3, 2.4)
Triglycerides	1.5 (0.6, 4.4)	1.6 (0.7, 4.2)	1.6 (0.7, 4.3)	1.3 (0.6, 3.9)	1.8 (0.7, 5.3)

1 Continuous variables are reported as geometric mean (95% prediction interval), and categorical variables are reported as counts (percentage). DMG, dimethylglycine; TMAO, trimethylamine-N-oxide; TML, trimethyllysine.

2 Defined according to self-reporting smoking habits and serum cotinine concentrations >85 nmol/L at baseline.

3 Defined according to self-reported, preexisting diagnosis.

4 Defined according to self-reported medication use.

5 *n* = number of participants with available results if different from 5746.

6 LDL concentration was calculated using the Friedewald equation (27).

7 Non-HDL-LDL cholesterol was calculated using the following formula: non-HDL-LDL cholesterol (mmol/L) = TC (mmol/L) – HDL cholesterol (mmol/L) – LDL cholesterol (mmol/L).

### Choline intake

Geometric mean (95% PI) energy-adjusted dietary choline intake for the total population was 260 (170,389) mg/d and was similar for all groups (**Table 3**). Dietary choline was mainly obtained in the form of phosphatidylcholine (44%), free choline (26%), and glycerophosphocholine (22%). Phosphocholine and sphingomyelin each contributed ∼5% to the total choline intake.

**TABLE 3:** Dietary intake among participants in the Hordaland Health Study 1997–1999^1^

		Elderly		Middle-aged	
Characteristic	Total population	Women	Men	Women	Men
*n* (%)	5746	1539 (26.8)	1247 (21.7)	1700 (29.6)	1260 (21.9)
Energy, kcal/d	1874 (946, 3359)	1527 (839, 2638)	1961 (1091, 3288)	1812 (1020, 2991)	2405 (1357, 3811)
Energy-adjusted choline intake,^2^ mg/d					
Total choline	260 (170, 389)	266 (189, 382)	258 (167, 390)	258 (176, 380)	256 (154, 402)
Free choline	68 (45, 102)	69 (49, 100)	67 (45, 102)	67 (46, 101)	68 (41, 108)
Glycerophosphocholine	54 (24, 108)	60 (34, 106)	56 (25, 109)	49 (24, 96)	53 (16, 118)
Phosphatidylcholine	111 (62, 198)	111 (67, 196)	109 (58, 202)	116 (71, 198)	107 (51, 196)
Phosphocholine	12 (5, 24)	13 (7, 24)	11 (5, 23)	12 (6, 25)	11 (3, 23)
Sphingomyelin	11 (6, 19)	12 (7, 18)	11 (6, 19)	12 (7, 18)	11 (5, 20)
Macronutrient intake,^3^ E%					
Carbohydrates	49.9 (38.5, 62.4)	51.7 (39.3, 65.2)	50.2 (40.1, 62.4)	49.2 (38.2, 61.8)	48.5 (37.6, 59.4)
Protein	15.8 (11.8, 21.0)	15.9 (11.8, 21.5)	15.7 (11.8, 21.0)	15.9 (11.8, 21.1)	15.4 (11.5, 20.4)
Fat	31.3 (20.6, 42.7)	29.9 (18.4, 42.4)	31.2 (20.9, 42.0)	32.0 (21.9, 43.4)	32.4 (23.1, 42.8)
MUFA	9.8 (6.1, 13.9)	9.2 (5.4, 13.7)	9.7 (6.1, 13.5)	10.1 (6.5, 13.8)	10.4 (7.3, 14.1)
PUFA	6.5 (3.8, 11.4)	5.9 (3.4, 10.2)	6.5 (3.9, 11.1)	6.7 (4.0, 11.9)	7 (4.2, 12.0)
SFA	12.2 (7.4, 18.2)	11.9 (6.7, 18.9)	12.1 (7.5, 18.4)	12.4 (7.9, 17.5)	12.3 (8.4, 17.3)
Alcohol	0.1 (0.0, 6.9)	0.0 (0.0, 5.1)	0.1 (0.0, 8.1)	0.2 (0.0, 6.1)	0.8 (0.0, 7.9)
Intake major food groups,^3^ g/1000 kcal					
Dairy	141 (23, 430)	172 (27, 492)	143 (20, 432)	121 (24, 387)	135 (20, 402)
Drinks	392 (122, 1087)	381 (102, 1090)	323 (93, 897)	460 (160, 1203)	398 (124, 933)
Eggs	5 (0, 25)	5 (0.0, 30)	5 (0, 29)	6 (1, 23)	5 (0, 20)
Fats	12 (2, 33)	11 (2, 31)	13 (3, 32)	13 (2, 33)	14 (3, 35)
Fish	35 (8, 104)	35 (8, 110)	44 (14, 115)	32 (9, 93)	31 (8, 87)
Fruit	98 (16, 334)	120 (22, 398)	86 (11, 297)	111 (22, 347)	74 (12, 263)
Grain products	118 (64, 192)	119 (61, 200)	118 (63, 195)	116 (660, 180)	120 (65, 184)
Meat	41 (10, 100)	31 (6, 83)	36 (10, 94)	49 (15, 106)	50 (16, 106)
Vegetables	142 (40, 368)	155 (26, 381)	145 (26, 350)	149 (49, 379)	118 (44, 302)
Sweets and snacks	1 (0, 27)	1 (0, 25)	1 (0, 25)	2 (0, 32)	2 (0, 26)

1 All dietary intakes are presented as geometric mean (95% prediction interval). E%, energy percentage.

2 Choline intake was energy-adjusted using the residual method (31).

3 Intake of macronutrients and major food groups was energy-adjusted using the density method (30).

**FIGURE 1:**
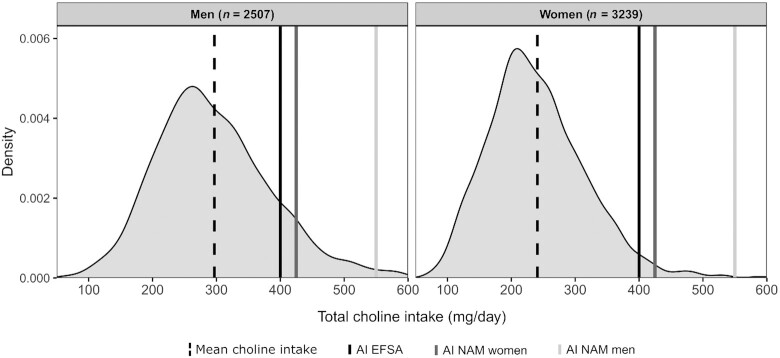
Distribution of mean choline intake for participants in the Hordaland Health Study 1997–1999. The solid black line indicates the AI for choline determined by EFSA (400 mg/d). The solid dark grey line indicates the AI for choline determined by NAM for women (425 mg/d), whereas the solid light grey line indicates this for men (550 mg/d). The y-axis shows the density of the observed values calculated using the kernel density estimation (32, 33). AI, adequate intake; EFSA, European Food Safety Authority; NAM, National Academy of Medicine.

Among women, 3% of both elderly and middle-aged participants had a non-energy-adjusted choline intake above EFSA's recommendation of 400 mg/d, whereas 7.5% elderly men achieved this AI (**Figure 1**). Around 19% of the middle-aged men achieved this AI. However, this dropped to 2% when using the AI set by NAM (550 mg/d).

### Foods contributing to choline intake

**FIGURE 2:**
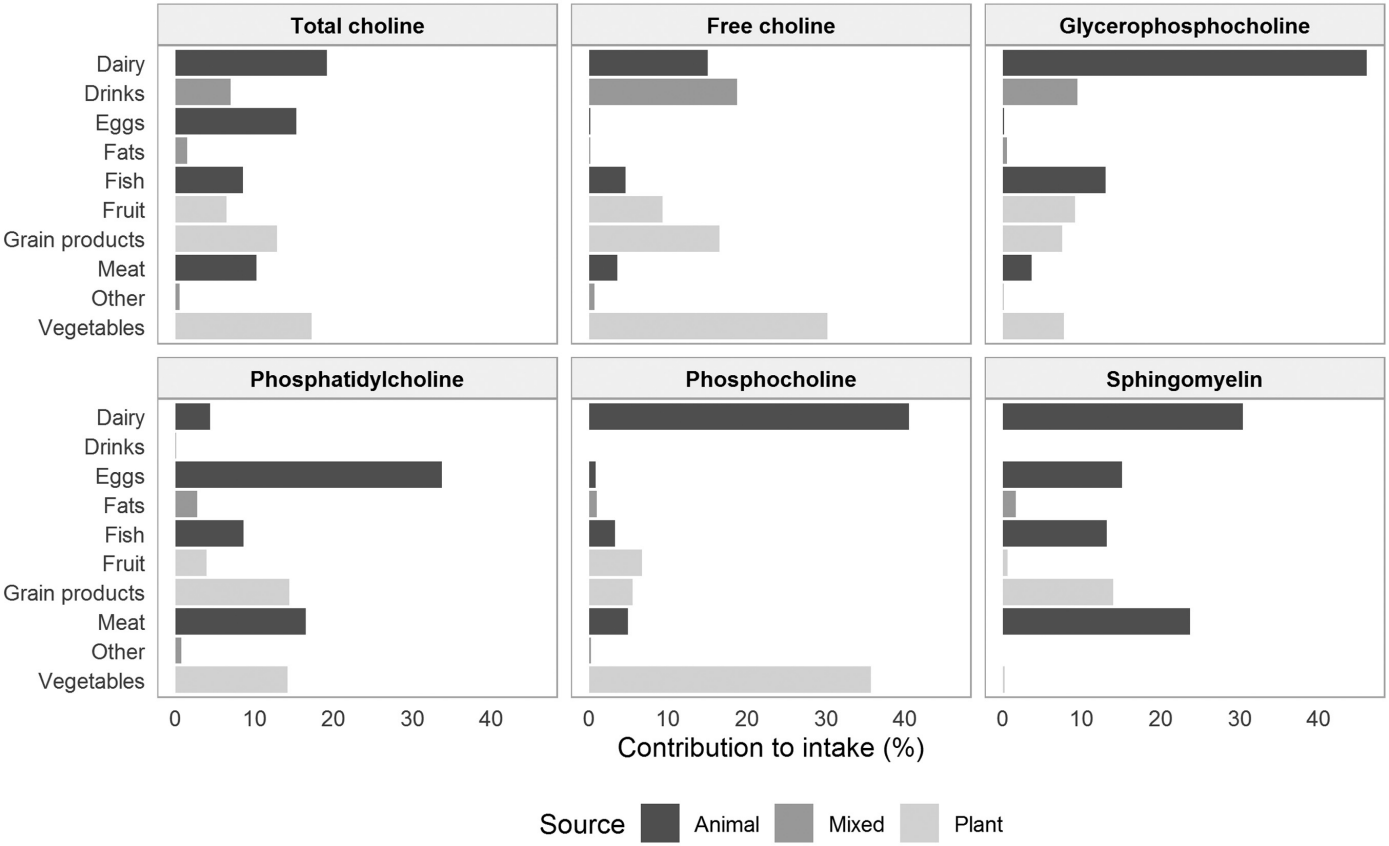
Overview of the main food groups from animal, plant, and mixed sources contributing to intake of total choline and individual choline forms for participants in the Hordaland Health Study 1997–1999. Contribution is indicated as percentage of total intake.

Dairy, vegetables, and eggs were the primary contributing food groups to total dietary choline intake in our study population (**Figure 2**). Main contributors differed largely between the different choline forms, with dairy being one of the most important sources of nearly all choline forms.

Besides food groups, we also investigated the food subcategories providing total choline and the individual choline forms. **Table 4** provides a list of the top 10 dietary total choline sources in our study population. Egg (15.3%) and low-fat milk (11.8%) consumption contributed with over one-fourth of the total dietary choline intake. Other, minor choline sources were potatoes, leafy vegetables, and whole-grain bread (respectively, 6.3%, 5.7%, and 5.2%). Contribution of food subcategories was calculated for each individual choline form, and results are presented in **Supplemental Tables 2–6**. Eggs were a major source of the lipid-soluble choline forms, whereas low-fat milk was a top-10 contributor to intake of all choline forms apart from PC.

**TABLE 4:** Primary food subcategories contributing to total choline intake among participants in the Hordaland Health Study 1997–1999

Rank	Food item	Contribution, %	Cumulative contribution, %
1	Eggs	15.3	15.3
2	Low-fat milk	11.8	27.1
3	Potatoes	6.3	33.4
4	Leafy vegetables	5.7	39.1
5	Whole-grain bread	5.2	44.3
6	Coffee	4.3	48.6
7	Fresh fruit	4.2	52.8
8	Meat spread	3.7	56.5
9	Other meat products	2.9	59.4
10	White bread	2.8	62.2

Contribution of the individual food items to the intake of total choline and the individual choline forms is presented in **Supplemental Table 7**.

### One-carbon metabolism

Plasma concentrations of one-carbon metabolites are shown in Table 2. Plasma concentrations of choline, DMG, cystathionine, and TML were similar across all groups. Men in both age groups had higher plasma betaine and methionine concentrations than women. However, the opposite was true for glycine and serine. Middle-aged participants had lower total homocysteine, cysteine, and TMAO plasma concentrations compared with elderly participants.

**FIGURE 3:**
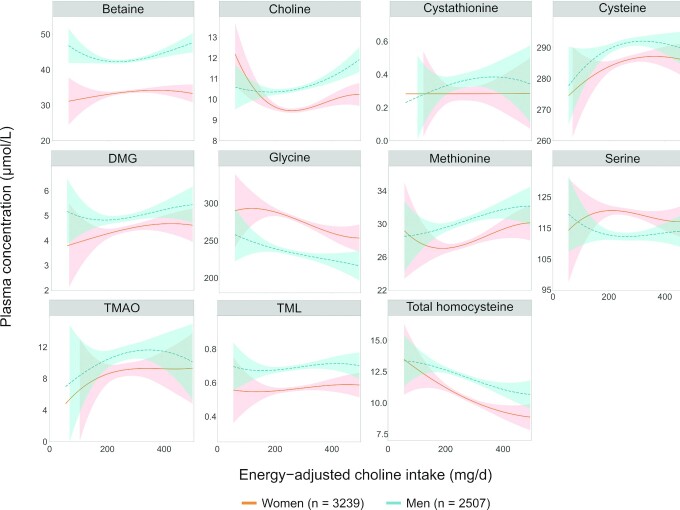
Associations between energy-adjusted choline intake and plasma concentration of one-carbon metabolites modeled as a polynomial spline in a model with sex as the interaction term and adjusted for age, BMI, and smoking for participants in the Hordaland Health Study 1997–1999. The dotted red and solid blue lines represent the modeled associations for women and men, respectively, and the colored areas indicate the corresponding 95% CI. DMG, dimethylglycine; TMAO, trimethylamine N-oxide; TML, trimethyllysine.

The relation between total energy–adjusted choline intake and plasma concentrations of one-carbon metabolites is shown in **Figure 3**. In women, plasma concentrations of choline and methionine showed a negative association with total choline intake at low intake levels, and the direction of the association changed at a choline intake of ±200 mg/d. The opposite was true for serine. Total choline intake was negatively associated with glycine and total homocysteine and positively with DMG. Furthermore, at low choline intakes, plasma cysteine and TMAO seemed to increase, whereas plasma concentrations flattened with increasing choline intake and even decreased at high choline intake for TMAO. Finally, no association was observed between dietary choline intake and betaine, cystathionine, or TML in women.

The association between choline intake and plasma concentrations of cysteine, glycine, methionine, TMAO, TML, and total homocysteine in men was similar to that in women. Plasma betaine showed a U-shaped association, whereas a positive association was observed for plasma choline and DMG, except at low intake levels, and for cystathionine until a choline intake of ±300 mg/d. Plasma serine was negatively associated with dietary choline intake, but the curve flattened at an intake of ±200 mg/d.

The associations of PC, free choline, phosphocholine, and sphingomyelin intake with plasma concentration of one-carbon metabolites were similar to that of total choline intake (**Supplemental Figures 1–4**). However, due to the low intake of phosphocholine and sphingomyelin, the 95% CI was rather large for all associations. Interestingly, we did not observe an association between free choline and plasma methionine, whereas we noted a positive association for the other choline forms and total choline. Finally, glycerophosphocholine was not associated with plasma concentrations of most of the one-carbon metabolites, but a weak positive association was observed for cysteine and TMAO (**Supplemental Figure 5**).

### Lipid metabolism

Plasma TC concentrations were similar in all groups apart from elderly women, who had a higher concentration (6.5 mmol/L) compared with the other groups (5.6–5.8 mmol/L). Both LDL and HDL cholesterol were also higher in elderly women compared with the rest of the population. In general, plasma cholesterol values were higher in women than men. Plasma TG values were similar in elderly women and men (1.8 mmol/L), whereas they were lower in middle-aged women and highest in middle-aged men (respectively, 1.4 mmol/L and 2.1 mmol/L).

**FIGURE 4:**
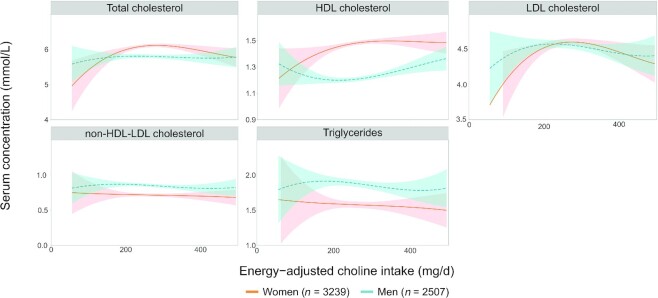
The association between energy-adjusted choline intake and serum concentration of lipid metabolites modeled as a polynomial spline in a model with sex as interaction term and adjusted for age, BMI, and smoking for participants in the Hordaland Health Study 1997–1999. The dotted red and solid blue lines represent the modeled associations for women and men, respectively, and the colored areas indicate the corresponding 95% CI.

The same model as for one-carbon metabolites was used to investigate the relation between total energy–adjusted choline intake and plasma concentrations of lipid metabolites (**Figure 4**). In women, total, HDL, and LDL cholesterol showed a positive association with choline intake until ±250 mg/d, whereafter the association became negative for TC and LDL cholesterol. Interestingly, this was not observed in men, and for plasma HDL at a low choline intake, a negative relation was found. In both men and women, a negative relation was found between choline intake and plasma TG, and no association was found with non-HDL-LDL cholesterol.

## Discussion

This study aimed to investigate the dietary intake of total choline and the individual choline forms, their dietary sources, and their association with one-carbon and lipid metabolites in community-dwelling Norwegian middle-aged and elderly adults. The mean energy-adjusted total choline intake was below the recommended AI set by both EFSA and NAM for women and men in both age groups. Choline was mainly consumed in the form of PC. The main foods contributing to total choline intake were eggs, low-fat milk, potatoes, leafy vegetables, and whole-grain bread. Food sources differed for the individual choline forms. Dietary choline intake was associated with plasma concentrations of one-carbon metabolites and with serum lipids.

### Dietary choline intake and sources

Dietary choline intake has been estimated in several cohorts globally (9, 11, 12–15, 36–41), and median intake has been estimated previously in our study population (42). In 8 European countries, the non-energy-adjusted estimated choline intakes in adults ranged from 269 to 468 mg/d (15). Similar findings have been observed in the United States, where estimated intakes ranged from 258 to 323 mg/d in women and 302 to 405 mg/d in men (9, 37–40). Interestingly, all studies drew the conclusion that most individuals do not achieve the AI for choline set by EFSA and NAM. The observed values of our cohort were rather low compared with previous findings. However, they are similar to self-reported dietary choline intake in a Norwegian population of patients with cardiovascular disease (43), using the same FFQ as in the current study. This could indicate that dietary choline intake is lower in Norway compared with other Western countries. Interestingly, a Swedish national dietary survey reported an estimated choline intake of 468 mg/d in men and 374 mg/d in women (15), which is higher than our findings. In this survey, dietary choline intake was estimated using a 4-d web-based food record, a method that differs greatly from the FFQ used in this study. The same USDA database was used for estimation of choline consumption. In addition, the reported choline intake was not energy adjusted, and dietary habits in Sweden differ from those in Norway. These factors might explain the observed difference in dietary choline intake. In general, choline intake estimates from various studies should be compared with caution due to substantial differences in method of dietary assessment, study population, and statistical methods.

In our study, the main source of dietary choline was PC, followed by free choline and glycerophosphocholine. The number of studies reporting on intake of individual choline forms is limited, but the available findings agree with ours (5, 14, 36, 44–46). This is not surprising because ∼60% of total choline in our study was obtained from animal products in which PC is the predominant form due to its incorporation in mammalian membranes (2). Indeed, several Western cohorts found eggs, meat, fish, and milk to be major contributors to total choline and thus PC intake (14, 36–40, 44). It should be mentioned that our data were gathered in 1997–1999, and available foods and dietary habits might have changed over time. Indeed, when comparing Norwegian consumption data from 1999 with data from 2018, we found a large increase in meat and egg consumption, a large decrease in milk consumption, and a small decrease in fish consumption (in 2018 compared with 2003 for fish consumption) (47). Dietary intake observed in our study population was comparable to the intake observed in the same age groups in the National Dietary Survey among Men and Women from 1997, supporting the external validity of our dietary data (48).

### Dietary choline and one-carbon metabolites

Our findings show that dietary choline intake is associated with the concentration of several metabolites involved in the one-carbon metabolism. Because nonfasting blood samples were analyzed, it must be considered that the concentration of metabolites could have been affected by prandial status. For example, it has been shown that plasma choline concentrations increase 10–15% after a meal and are particularly responsive to large intakes of dietary choline sources (16). Also, dietary choline intake in our study reflects the long-term intake, whereas plasma one-carbon metabolites were only measured at a single time point. Nevertheless, we observed that plasma choline was positively associated with self-reported long-term choline intake in men, whereas this association was unclear in women. It has previously been reported that dietary choline intake is not associated with plasma choline concentrations, in contrast with our findings (49, 50). However, it has been shown that plasma choline is associated with egg consumption, which was the main choline contributor in our study population (51). In general, it can be questioned whether plasma choline is a good biomarker of choline intake at all, because it is homeostatically regulated by mechanisms such as de novo synthesis. For example, fasting plasma choline concentrations did not alter in premenopausal women after a moderate increase in dietary choline intake during 12 wk (52).

We also observed a negative association between choline intake and plasma concentrations of total homocysteine in both men and women. Simultaneously, a positive association with methionine was observed. Indeed, the homocysteine-lowering effect of choline has been previously observed in several studies (17, 22, 36, 40) and is likely to be caused by methionine synthesis and remethylation of homocysteine through choline's metabolite betaine (53). Although plasma betaine concentration was not associated with dietary choline in our study population, a positive association was observed with DMG. A possible explanation could be that betaine is used in the remethylation of homocysteine to methionine and thus forms DMG. In addition, increased dietary choline intake leads to decreased choline de novo synthesis, a process that requires a high amount of methyl groups and therefore methionine (18). Thus, choline intake not only is methionine saving but also actively contributes to methionine synthesis via betaine and the betaine homocysteine methyltransferase (BHMT) pathway (2, 4). Supplementation of betaine has been used to lower homocysteine concentrations in hyperhomocysteinemia (54) and has been shown to decrease homocysteine concentrations by up to 20% in the general population (55). However, high doses of betaine supplementation have been associated with increased LDL cholesterol, whereas this association has not been observed with choline supplementation (22, 56). We did, however, find a positive association between dietary choline intake and serum LDL cholesterol concentrations but only up to an intake of ±250 mg/d.

Choline is a dietary precursor for TMAO, a compound that has been associated with risk of cardiovascular disease (CVD) (57). Indeed, a positive association was observed between dietary choline intake and plasma TMAO concentrations in our study, but only up to 300 mg/d. Afterward, plasma TMAO declined with increasing choline consumption. TMAO can be formed from choline-containing compounds, betaine, and L-carnitine after formation of trimethylamine by gut bacteria (58). Recently, red meat, fish, and egg intakes have been associated with serum TMAO concentrations (59), but results of several studies assessing the association between dietary intake (mainly of choline and egg) and circulating TMAO concentrations were inconclusive (60–63). In addition, the number of studies focusing on TMAO and CVD is increasing, but it remains unclear whether TMAO is a causative factor or a biomarker for CVD (64). In an earlier study, we found that TMAO did not mediate the association between dietary choline intake and risk of acute myocardial infarction in patients with stable angina pectoris (65). Furthermore, a recent review by Thomas and Fernandez (66) investigating the role of diet and TMAO in CVD concluded that further evaluation of how TMAO precursors influence CVD and plasma TMAO concentrations is needed.

Sex differences in the association between choline intake and one-carbon metabolite concentrations have been observed. Indeed, several enzymes are involved in the one-carbon metabolism or up- or downregulated in women compared with men. Sadre-Marandi et al. (67) identified 5 enzymes—BHMT, methionine synthase (MS), methylenetetrahydrofolate reductase (MTHFR), serine hydroxymethyltransferase (SHMT), and phosphatidylethanolamine-N-methyltransferase (PEMT)—with a large difference in expression between women and men. For PEMT, BHMT, and MTHFR, sex hormones are thought to be at the base of the discrepancies in expression and/or activity, whereas the cause remains unclear for MS and SHMT. Subgroup analyses have a high risk of bias, and although their importance in exploratory analyses has been highlighted (68), our results are hypothesis generating and need to be confirmed in further research. In addition, interindividual differences in one-carbon metabolite response to dietary choline should be taken into account. The one-carbon metabolism is regulated by a wide range of enzymes and nutritional factors that may contribute to interindividual differences in the response to choline intake. For example, several of the involved enzymes are encoded by genes containing single-nucleotide polymorphisms, leading to alterations in gene expression or enzyme activity, choline requirement, and possibly metabolite response (21). Finally, the within-person reproducibility of most investigated metabolites is fair to excellent [intraclass correlation coefficient (ICC): >0.75–0.40], except for methionine, choline, and TML (ICC: 0.39–0.15), to allow one-exposure assessment of biomarker status in epidemiologic studies (69).

### Dietary choline and lipid metabolites

Effects of dietary choline on serum lipid metabolites remain poorly studied, and findings are inconclusive. Consumption of 3 eggs a day for 4 wk increased total, HDL, and LDL cholesterol in healthy volunteers compared with choline bitartrate supplementation (70). Similar findings were observed in Ldlr^–/–^ male mice after being fed a diet enriched with PC but not free choline (71). However, PC supplementation for 2 wk in 26 healthy volunteers did not alter cholesterol concentration but did increase serum TGs (56). These findings indicate that the choline form might be important when measuring the effect on lipid metabolites. Furthermore, PC was the main consumed choline form, and eggs were its main food source in this study. Because eggs contain a high amount of cholesterol (72), it is not unlikely that the observed short-term increase in serum total and LDL cholesterol might be caused by the composition of the food item providing choline rather than by choline itself. Notably, statin use was not widespread at the time of data collection. When comparing our findings with contemporary populations of the same age, it should thus be kept in mind that reported TC concentrations reflect untreated cholesterol concentrations.

### Strengths and limitations

The main advantage of this study is the large sample size of community-dwelling adults. In addition, we have investigated the dietary intake and contributors of individual choline forms, something that is understudied. Furthermore, the FFQ used in this study captured long-term food intake and dietary patterns over time and avoided day-to-day variation in dietary intake (73). It is a method with low cost and low participant burden and is therefore frequently used for large cohort studies (73).

However, several limitations of this study should be acknowledged. Unfortunately, the FFQ used to estimate dietary intake was not validated for choline intake, which did not allow us to evaluate how well it captured the actual choline intake. Inherent limitations of an FFQ, such as systematic measurement errors, also apply to this study and may have led to a systematic underestimation of actual choline intakes (74). Furthermore, the calculation of the choline content of food items was based on the USDA database (29) because the Norwegian food composition table does not include values for choline (www.matvaretabellen.no). As choline content of food items may differ due to season and geographical location and variation between and within choline sources, the choline value obtained from the USDA database might not always reflect the true choline content of food items (29). This is especially an issue for local foods, which may not typically be consumed in a North American diet. In general, choline intake data from food items are limited, and actual intakes may be underestimated because the choline content of only a small number of foods has been investigated. In addition, choline intakes from food additives such as lecithin cannot be estimated accurately as per now. Considering the increasing consumption of processed foods, it is not unlikely that a significant amount of dietary choline intake is derived from these sources, which leads again to an underestimation of choline consumption in current studies. Finally, the FFQ did not cover possible choline intake from supplements. However, most mainstream multivitamin supplements do not contain choline, especially not at the time of our data collection (1997–1999), and findings from the United States suggest that very few consume choline as a single-nutrient supplement (9). Moreover, it has been suggested that multivitamin or mineral supplement intake does not affect choline intake at the population level (75).

### Conclusions

In this study including community-dwelling Norwegian middle-aged and elderly adults, we observed that the self-reported intake of dietary choline was below the established AI for most participants. Furthermore, choline was mainly consumed in the form of PC, and major contributing dietary sources were eggs, low-fat milk, potatoes, and leafy vegetables. Dietary choline was associated with the plasma concentration of one-carbon metabolites and serum lipids.

Further studies that estimate the choline intake in Nordic populations are warranted to allow for the establishment of dietary recommendations and nutrition policy. Also, because this is an exploratory study, there is a need to clarify the association between dietary choline, especially the individual choline forms, and one-carbon and lipid metabolites as these are closely related to risk of chronic diseases.

## Supplementary Material

nxab367_Supplemental_File

## Data Availability

Data described in the manuscript, code book, and analytical code will be made available upon request pending application and approval.

## Notes

The authors reported no funding received for this study.

Author disclosures: The author reports no conflicts of interest.

Supplemental Figures 1–5 and Supplemental Tables 1–7 are available from the “Supplementary data” link in the online posting of the article and from the same link in the online table of contents at https://academic.oup.com/jn/.

Abbreviations used: AI, adequate intake; BHMT, betaine homocysteine methyltransferase; CVD, cardiovascular disease; DMG, dimethylglycine; EFSA, European Food Safety Authority; E%, energy percentage; HUSK, Hordaland Health Study; ICC, intraclass correlation coefficient; MS, methionine synthase; MTHFR, methylenetetrahydrofolate reductase; NAM, National Academy of Medicine; PC, phosphatidylcholine; PEMT, phosphatidylethanolamine-N-methyltransferase; PI, prediction interval; SHMT, serine hydroxymethyltransferase; TC, total cholesterol; TG, triglyceride; TMAO, trimethylamine-N-oxide; TML, trimethyllysine.
