# Supplementary material for: Assessment of Dietary Choline Intake, Contributing Food Items, and Associations with One-Carbon and Lipid Metabolites in Middle-Aged and Elderly Adults: The Hordaland Health Study
Source: J Nutr. 2021 Oct 13;152(2):513–24. doi: 10.1093/jn/nxab367 (PMC8826836; doi:10.1093/jn/nxab367)
Supplement: nxab367_Supplemental_File [file nxab367_supplemental_file.zip › Supplementary Figures.docx]

**
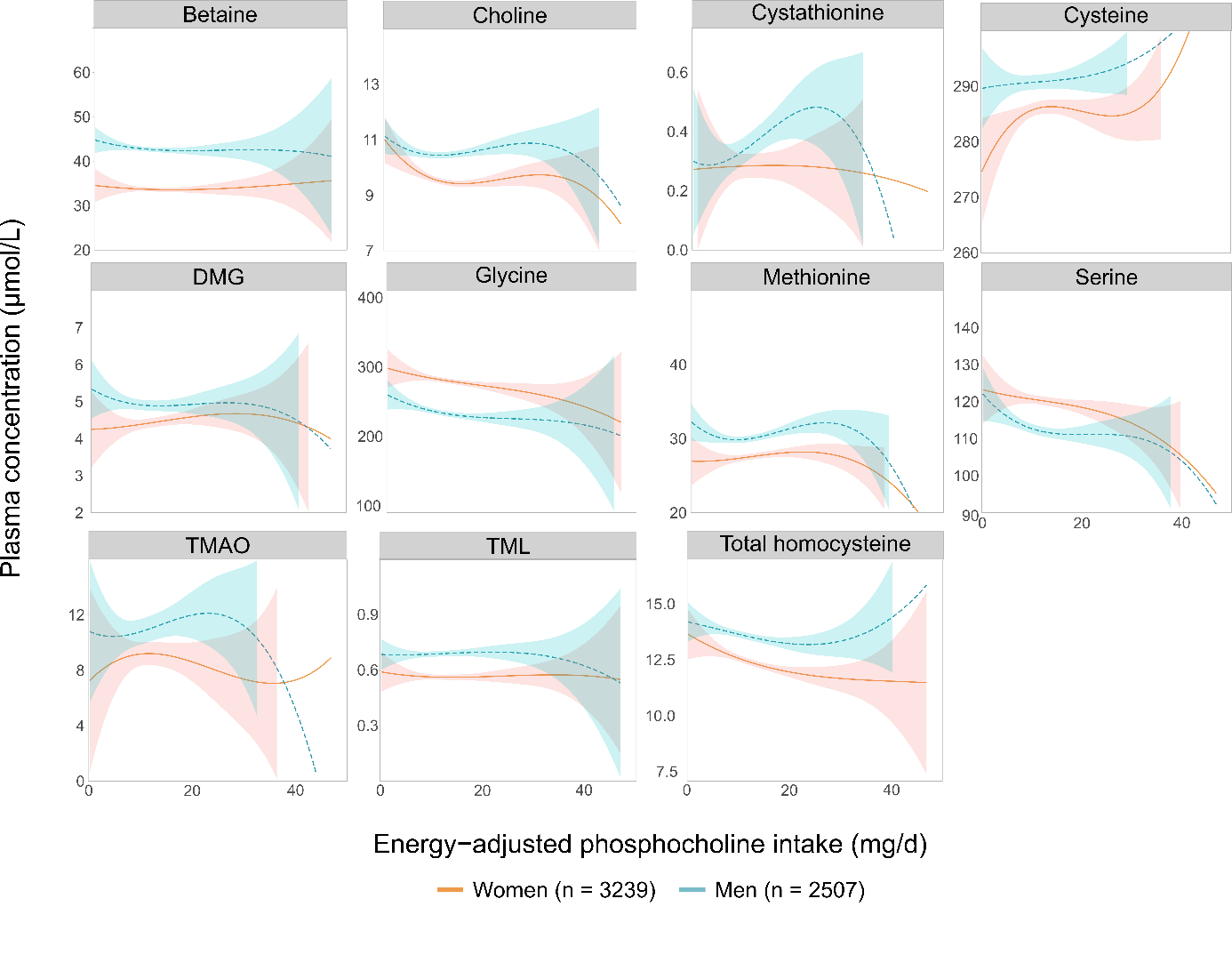
**

**Supplementary Figure 1**: Associations between energy-adjusted phosphatidylcholine intake and plasma concentration of one-carbon metabolites modelled as a polynomial spline in a model with sex as interaction term and adjusted for age, BMI, and smoking for participants in the Hordaland Health Study 1997-1999. The dotted red and solid blue lines represent the modelled associations for women and men respectively, and the colored areas indicate the corresponding 95% confidence interval. DMG indicates dimethylglycine; TMAO, trimethylamine N-oxide; TML, trimethyllysine.

**
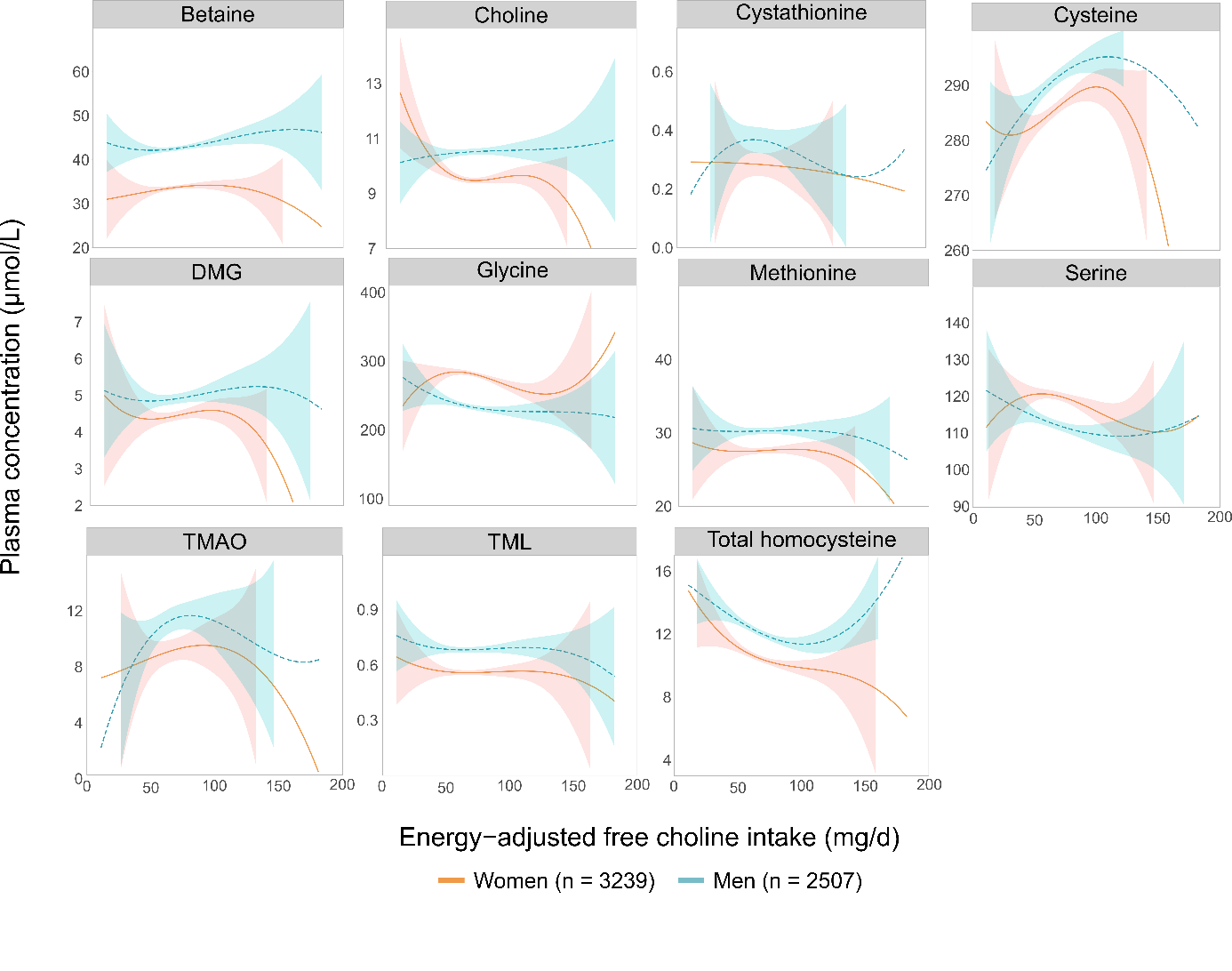
**

**Supplementary Figure 2**: Associations between energy-adjusted free choline intake and plasma concentration of one-carbon metabolites modelled as a polynomial spline in a model with sex as interaction term and adjusted for age, BMI, and smoking for participants in the Hordaland Health Study 1997-1999. The dotted red and solid blue lines represent the modelled associations for women and men respectively, and the colored areas indicate the corresponding 95% confidence interval. DMG indicates dimethylglycine; TMAO, trimethylamine N-oxide; TML, trimethyllysine.


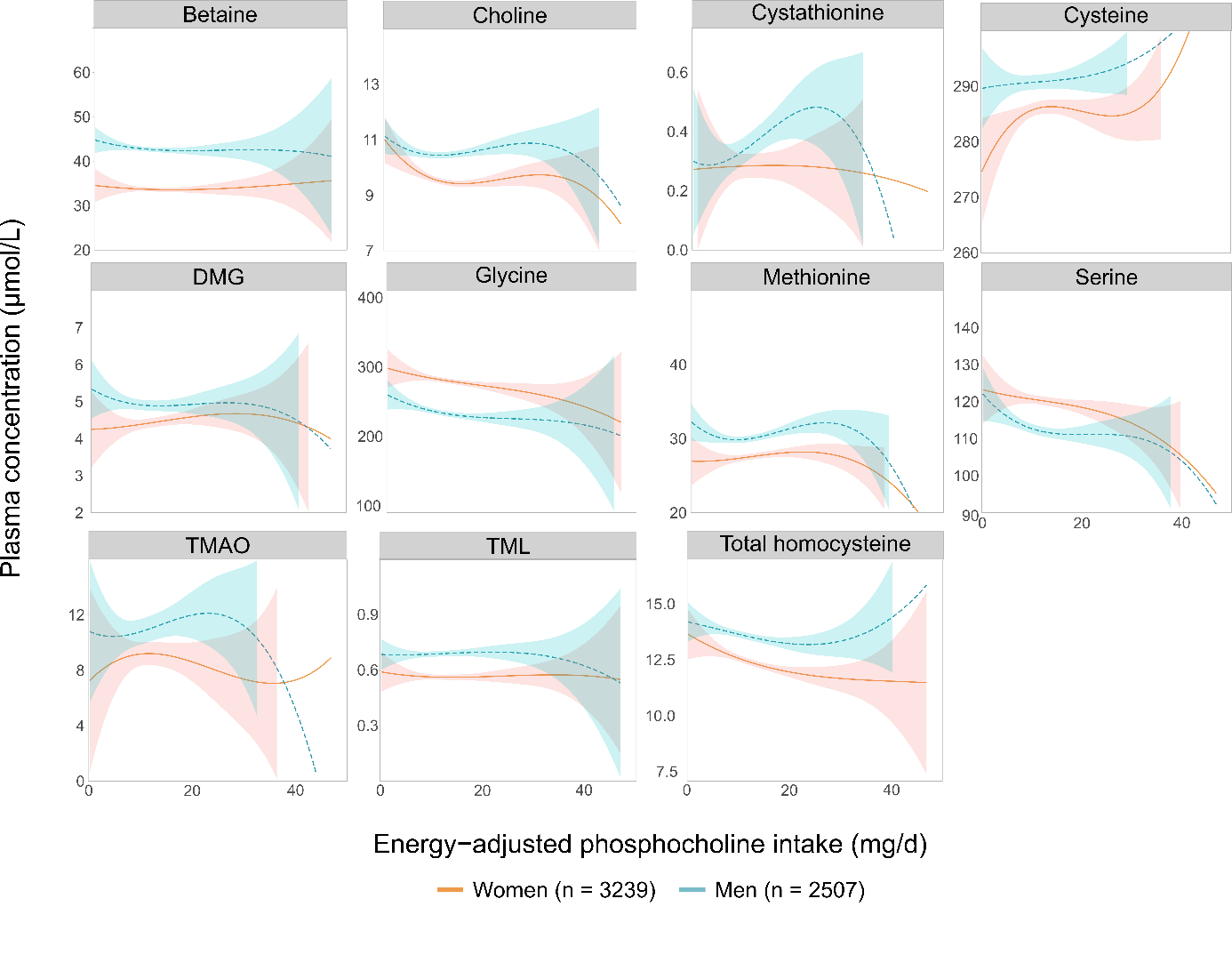


**Supplementary Figure 3**: Associations between energy-adjusted phosphocholine intake and plasma concentration of one-carbon metabolites modelled as a polynomial spline in a model with sex as interaction term and adjusted for age, BMI, and smoking for participants in the Hordaland Health Study 1997-1999. The dotted red and solid blue lines represent the modelled associations for women and men respectively, and the colored areas indicate the corresponding 95% confidence interval. DMG indicates dimethylglycine; TMAO, trimethylamine N-oxide; TML, trimethyllysine.

**
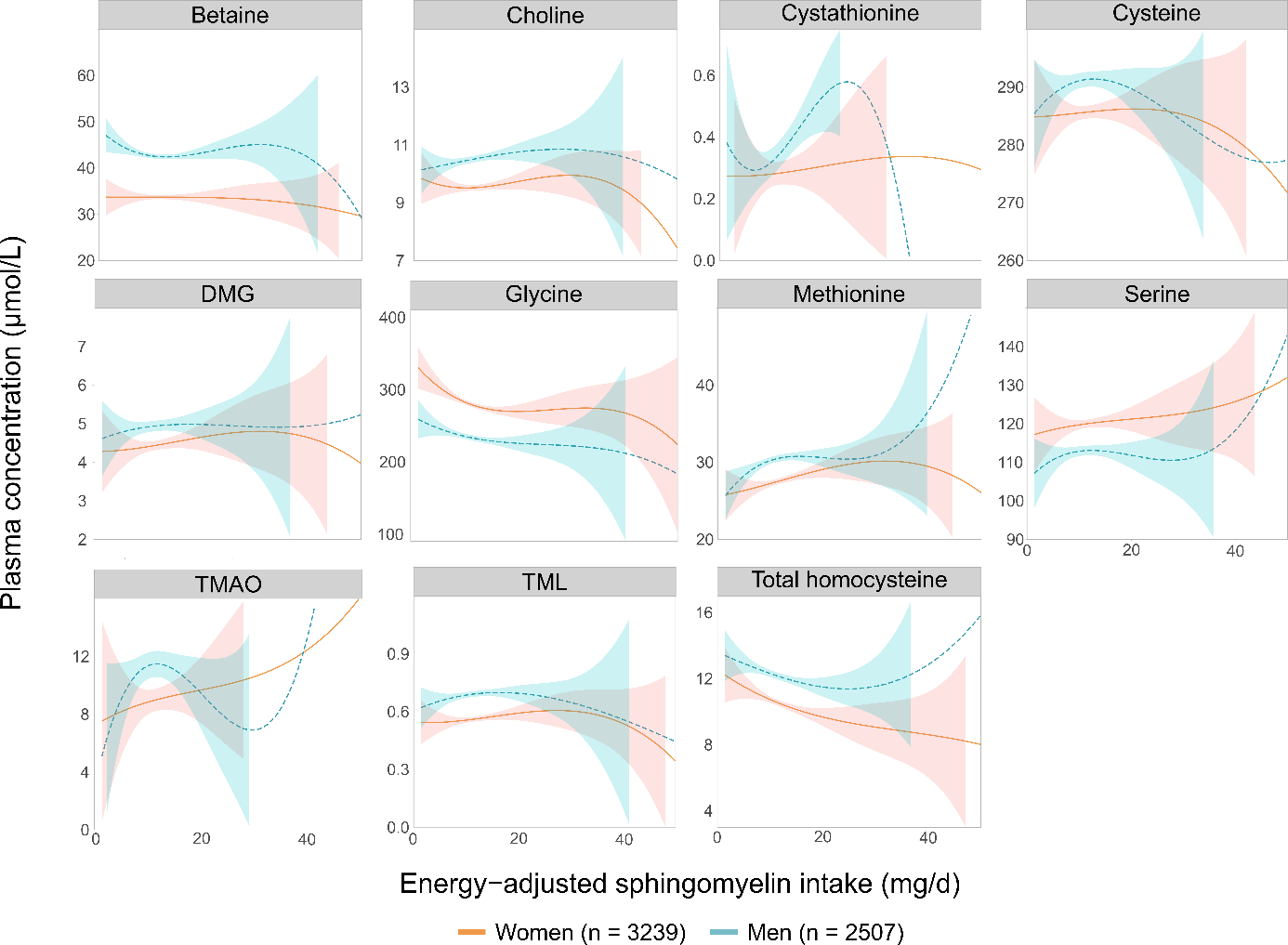
**

**Supplementary Figure 4**: Associations between energy-adjusted sphingomyelin intake and plasma concentration of one-carbon metabolites modelled as a polynomial spline in a model with sex as interaction term and adjusted for age, BMI, and smoking for participants in the Hordaland Health Study 1997-1999. The dotted red and solid blue lines represent the modelled associations for women and men respectively, and the colored areas indicate the corresponding 95% confidence interval. DMG indicates dimethylglycine; TMAO, trimethylamine N-oxide; TML, trimethyllysine.


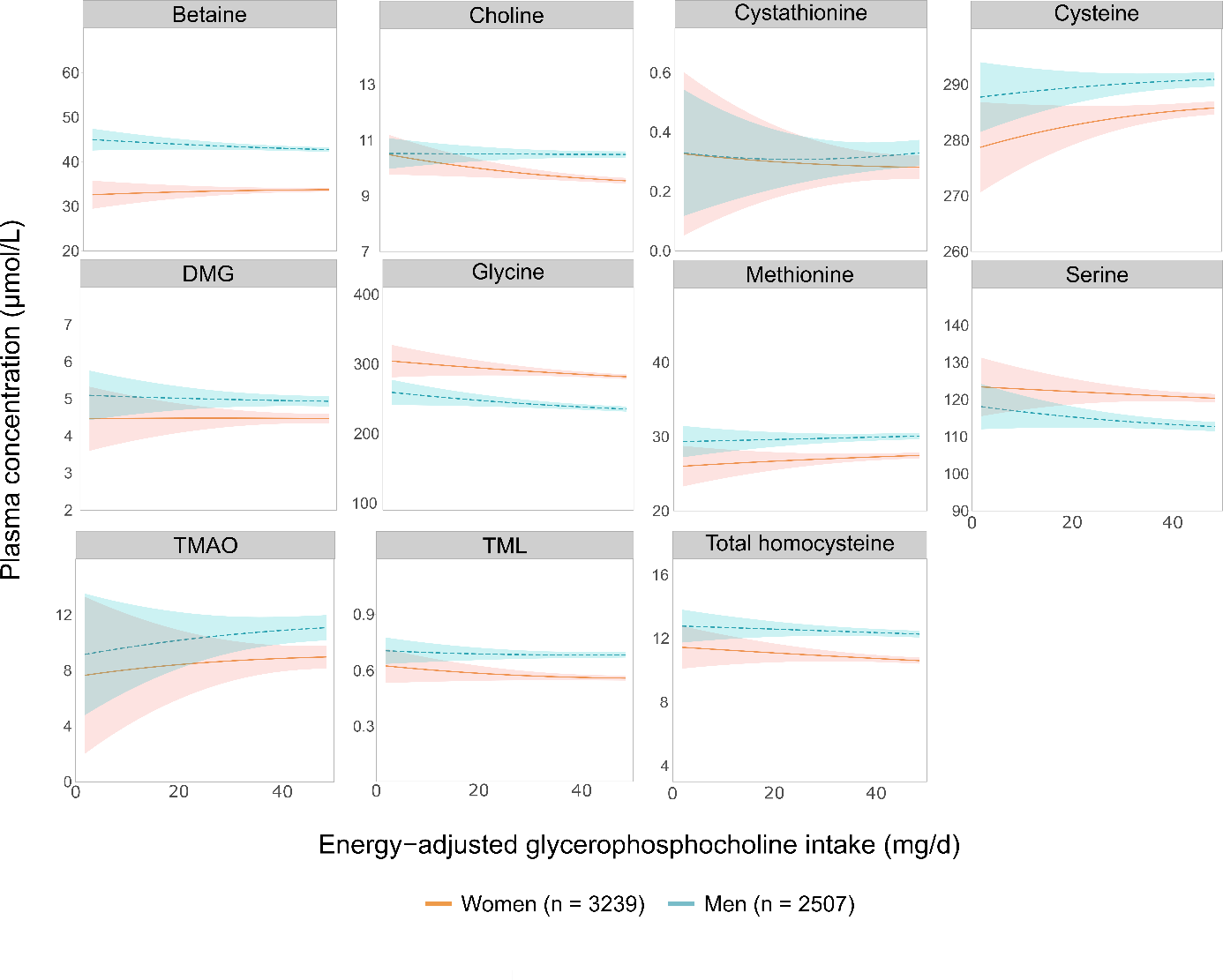


**Supplementary Figure 5**: Associations between energy-adjusted glycerophosphocholine intake and plasma concentration of one-carbon metabolites modelled as a polynomial spline in a model with sex as interaction term and adjusted for age, BMI, and smoking for participants in the Hordaland Health Study 1997-1999. The dotted red and solid blue lines represent the modelled associations for women and men respectively, and the colored areas indicate the corresponding 95% confidence interval. DMG indicates dimethylglycine; TMAO, trimethylamine N-oxide; TML, trimethyllysine.
