# Supplementary material for: Assessment of Dietary Choline Intake, Contributing Food Items, and Associations with One-Carbon and Lipid Metabolites in Middle-Aged and Elderly Adults: The Hordaland Health Study
Source: J Nutr. 2021 Oct 13;152(2):513–24. doi: 10.1093/jn/nxab367 (PMC8826836; doi:10.1093/jn/nxab367)
Supplement: nxab367_Supplemental_File [file nxab367_supplemental_file.zip › Supplementary Tables.docx]

| **Supplementary Table 1:** Grouping of food items into subcategories, categories, and food groups | | | |
| --- | --- | --- | --- |
| **Food group** | **Category** | **Subcategory** | **Food items** |
|  |  |  |  |
| Dairy | Milk | Whole milk | Whole milk |
|  |  | Low-fat milk | Low-fat milk |
|  |  |  | Skimmed milk |
|  |  | Other milk | Unspecified milk |
|  | Cheese | White cheese | White cheese, full-fat |
|  |  |  | White cheese, semi-fat |
|  |  | Brown cheese | Brown cheese |
|  | Other dairy | Yoghurt | Yoghurt |
|  |  | Cream | Cream |
|  |  |  | Sour cream |
|  |  | Ice cream | Ice cream |
|  |  | Other dairy products | Other dairy products |
| Drinks | Coffee |  | Coffee |
|  | Tea |  | Tea |
|  | Soda | Soda sugar | Soda with sugar |
|  |  |  | Lemonade with sugar |
|  |  | Soda light | Soda light |
|  |  |  | Lemonade light |
|  | Alcohol | Beer | Beer |
|  |  |  | Alcohol-free beer |
|  |  | Wine | Wine |
|  |  | Liquor | Liquor |
| Eggs | Eggs |  | Eggs |
| Fats | Margarine |  | Soya margarine |
|  |  |  | Margarine mixture |
|  |  |  | Other margarine |
|  |  |  | Light margarine |
|  | Butter |  | Butter |
|  |  |  | Butter-margarine mixture |
|  |  |  | Unspecified butter |
| Fish | Fatty fish |  | Salmon |
|  |  |  | Trout |
|  |  |  | Other fatty fish |
|  | Fish products | Fish spread | Fish spread |
|  |  | Other fish product | Other fish product |
|  | Shellfish |  | Shellfish |
| Fruit | Fresh fruit |  | Citrus fruit |
|  |  |  | Apple |
|  |  |  | Pear |
|  |  |  | Unspecified fruit |
|  | Juice |  | Juice |
|  | Other fruit | Conserved fruit/berries | Jam |
|  |  |  | Marmalade |
|  |  |  | Canned fruit |
| Grain products | Bread | White bread | White bread |
|  |  |  | Bread <50% whole wheat |
|  |  | Wholegrain bread | Bread >50% whole wheat |
|  |  | Other bread | "Lefse" |
|  |  |  | Chapatti |
|  |  |  | Taco |
|  |  |  | Flatbread |
|  |  |  | Crispbread |
|  | Pastries | Buns | Waffles |
|  |  |  | Yeast baked goods |
|  |  | Biscuits | Biscuits |
|  |  | Other pastries | Other pastries |
|  | Other grain products | Pizza | Pizza |
|  |  | Cereal | Cereal |
|  |  |  | Oatmeal |
| Meat | Fresh/frozen meat | Poultry | Poultry |
|  |  | Other fresh meat | Unspecified fresh meat |
|  | Meat products | Meat spread | Liver spread |
|  |  |  | Other meat spread |
|  |  | Other meat products | Other meat products |
| Sweets and snacks | Sugar and sweets | Sugar | Sugar |
|  |  |  | Other sweeteners |
|  |  | Sweet spread | Sweat spread |
|  |  | Sweets | Chocolate |
|  |  |  | Candy |
|  | Snacks | Chips | Potato chips |
|  |  | Nuts and seeds | Nuts and seeds |
|  |  | Other snacks | Other snacks |
| Vegetables | Potatoes |  | French fries |
|  |  |  | Fresh potatoes |
|  | Fresh vegetables | Root vegetables | Carrot |
|  |  |  | Turnip |
|  |  | Leafy vegetables | Cabbage |
|  |  |  | Cauliflower |
|  |  |  | Broccoli |
|  |  |  | Spinach |
|  |  |  | Parsley |
|  |  | Other vegetables | Leek |
|  |  |  | Onion |
|  |  |  | Tomato |
|  |  |  | Bell pepper |
|  |  |  | Vegetable mix |
|  |  |  | Unspecified vegetables |
|  | Canned vegetables |  | Pickled vegetables |
|  |  |  | Other canned vegetables |

| **Supplementary Table 2**: Primary food subcategories contributing to free choline intake among participants in the Hordaland Health Study 1997-1999 | | | |
| --- | --- | --- | --- |
| **Rank** | **Food item** | **Contribution (%)** | **Cumulative contribution (%)** |
| 1 | Coffee | 12.1 | 12.1 |
| 2 | Potatoes | 12.0 | 24.1 |
| 3 | Low fat milk | 8.7 | 32.8 |
| 4 | Wholegrain bread | 8.2 | 41.0 |
| 5 | Leafy vegetables | 8.2 | 49.2 |
| 6 | Fresh fruit | 6.8 | 56.0 |
| 7 | Root vegetables | 5.4 | 61.4 |
| 8 | Alcohol | 5.2 | 66.6 |
| 9 | White bread | 4.5 | 71.1 |
| 10 | Other vegetables | 3.8 | 74.9 |

| **Supplementary Table 3**: Primary food subcategories contributing to glycerophosphocholine intake among participants in the Hordaland Health Study 1997-1999 | | | |
| --- | --- | --- | --- |
| **Rank** | **Food item** | **Contribution (%)** | **Cumulative contribution (%)** |
| 1 | Low fat milk | 31.7 | 31.7 |
| 2 | Fatty fish | 5.5 | 37.2 |
| 3 | Potatoes | 5.4 | 42.6 |
| 4 | Coffee | 5.0 | 47.6 |
| 5 | Fresh fruit | 4.9 | 52.5 |
| 6 | Alcohol | 3.9 | 56.4 |
| 7 | Yoghurt | 3.7 | 60.1 |
| 8 | Whole milk | 3.6 | 63.7 |
| 9 | Wholegrain bread | 3.3 | 67.0 |
| 10 | Other milk | 3.3 | 70.3 |

| **Supplementary Table 4**: Primary food subcategories contributing to phosphatidylcholine intake among participants in the Hordaland Health Study 1997-1999 | | | |
| --- | --- | --- | --- |
| **Rank** | **Food item** | **Contribution (%)** | **Cumulative contribution (%)** |
| 1 | Eggs | 33.8 | 33.8 |
| 2 | Meat spread | 6.3 | 40.1 |
| 3 | Leafy vegetables | 6.2 | 46.3 |
| 4 | Wholegrain bread | 4.7 | 51.0 |
| 5 | Other meat products | 4.4 | 55.4 |
| 6 | Potatoes | 4.2 | 59.6 |
| 7 | Buns | 3.8 | 63.4 |
| 8 | Poultry | 3.5 | 66.9 |
| 9 | Shellfish | 3.2 | 70.1 |
| 10 | Fresh fruit | 2.7 | 72.8 |

| **Supplementary Table 5**: Primary food subcategories contributing to phosphocholine intake among participants in the Hordaland Health Study 1997-1999 | | | |
| --- | --- | --- | --- |
| **Rank** | **Food item** | **Contribution (%)** | **Cumulative contribution (%)** |
| 1 | Low fat milk | 26.7 | 26.7 |
| 2 | Leafy vegetables | 15.1 | 41.8 |
| 3 | Potatoes | 8.6 | 50.4 |
| 4 | Other vegetables | 6.3 | 56.7 |
| 5 | Root vegetables | 5.2 | 61.9 |
| 6 | Whole milk | 4.2 | 66.1 |
| 7 | Fresh fruit | 4.0 | 70.1 |
| 8 | Yoghurt | 3.7 | 73.8 |
| 9 | Other milk | 2.8 | 76.6 |
| 10 | Poultry | 2.1 | 78.7 |

| **Supplementary Table 6**: Primary food subcategories contributing to sphingomyelin intake among participants in the Hordaland Health Study 1997-1999 | | | |
| --- | --- | --- | --- |
| **Rank** | **Food item** | **Contribution (%)** | **Cumulative contribution (%)** |
| 1 | Eggs | 15.1 | 15.1 |
| 2 | Low fat milk | 12.2 | 27.3 |
| 3 | Poultry | 9.7 | 37.0 |
| 4 | White cheese | 6.4 | 43.4 |
| 5 | Wholegrain bread | 6.4 | 49.8 |
| 6 | Meat spread | 6.1 | 55.9 |
| 7 | Other meat products | 5.4 | 61.3 |
| 8 | Brown cheese | 3.5 | 64.8 |
| 9 | Shellfish | 3.1 | 67.9 |
| 10 | White bread | 2.9 | 70.8 |

| **Supplementary Table 7:** Main food groups, categories, and subcategories contributing to intake of total choline and individual choline forms among participants in the Hordaland Health Study 1997-1999 | | | | | | | | | | |
| --- | --- | --- | --- | --- | --- | --- | --- | --- | --- | --- |
|  | | | | |  | **Water-soluble forms** | | | **Lipid-soluble forms** | |
| **Contributing foods (%)** | | | | | **Total choline** | **Free choline** | **Glycerophosphocholine** | **Phosphocholine** | **Phosphatidylcholine** | **Sphingomyelin** |
| Dairy | | | | | 19.2 | 15.0 | 46.1 | 40.5 | 4.4 | 30.4 |
|  | | Milk | | | 14.5 | 11.1 | 38.6 | 33.7 | 2.1 | 15.2 |
|  | |  | | Low-fat milk | 11.8 | 8.7 | 31.7 | 26.7 | 1.7 | 12.2 |
|  | |  | | Whole milk | 1.5 | 1.5 | 3.6 | 4.2 | 0.1 | 1.7 |
|  | |  | | Other milk | 1.2 | 0.9 | 3.3 | 2.8 | 0.2 | 1.3 |
|  | | Cheese | | | 1.9 | 1.8 | 1.2 | 1.3 | 1.5 | 9.9 |
|  | |  | | White cheese | 1.3 | 1.2 | 1.1 | 1.3 | 0.9 | 6.4 |
|  | |  | | Brown cheese | 0.6 | 0.7 | 0.1 | 0.0 | 0.5 | 3.5 |
|  | | Other dairy | | | 2.8 | 2.1 | 6.3 | 5.4 | 0.8 | 5.3 |
|  | |  | | Yoghurt | 1.5 | 1.0 | 3.7 | 3.7 | 0.4 | 2.6 |
|  | |  | | Cream | 0.5 | 0.4 | 1.0 | 0.6 | 0.2 | 1.4 |
|  | |  | | Ice cream | 0.6 | 0.4 | 1.3 | 0.8 | 0.2 | 1.3 |
|  | |  | | Other dairy products | 0.2 | 0.3 | 0.3 | 0.3 | 0.0 | 0.0 |
|  | | | | | | | | | | |
| Drinks | | | | | 7.0 | 18.7 | 9.5 | 0.0 | 0.0 | 0.0 |
|  | | Coffee | | | 4.3 | 12.1 | 5.0 | 0.0 | 0.0 | 0.0 |
|  | | Tea | | | 0.3 | 1.3 | 0.0 | 0.0 | 0.0 | 0.0 |
|  | | Soda | | | 0.2 | 0.1 | 0.5 | 0.0 | 0.1 | 0.0 |
|  | |  | | Soda sugar | 0.2 | 0.1 | 0.5 | 0.0 | 0.1 | 0.0 |
|  | |  | | Soda light | 0.0 | 0.0 | 0.0 | 0.0 | 0.0 | 0.0 |
|  | | Alcohol | | | 2.2 | 5.2 | 3.9 | 0.0 | 0.0 | 0.0 |
|  | |  | | Beer | 1.8 | 3.9 | 3.4 | 0.0 | 0.0 | 0.0 |
|  | |  | | Wine | 0.5 | 1.3 | 0.5 | 0.0 | 0.0 | 0.0 |
|  | |  | | Liquor | 0.0 | 0.0 | 0.0 | 0.0 | 0.0 | 0.0 |
|  | | | | | | | | | | |
| Eggs | | | | | 15.3 | 0.2 | 0.2 | 0.8 | 33.8 | 15.1 |
|  | | | | | | | | | | |
| Fats | | | | | 1.5 | 0.2 | 0.6 | 1.0 | 2.8 | 1.7 |
|  | | Margarine | | | 0.3 | 0.0 | 0.1 | 0.2 | 0.7 | 0.0 |
|  | | Butter | | | 0.2 | 0.0 | 0.1 | 0.2 | 0.2 | 0.9 |
|  | | | | | | | | | | |
| Fish | | | | | 8.6 | 4.6 | 13.0 | 3.3 | 8.7 | 13.2 |
|  | | Fatty fish | | | 2.1 | 1.7 | 5.5 | 0.4 | 0.8 | 2.3 |
|  | | Fish products | | | 4.8 | 3.0 | 7.0 | 2.5 | 4.7 | 7.8 |
|  | |  | | Fish spread | 3.5 | 2.0 | 4.7 | 1.5 | 3.8 | 6.4 |
|  | |  | | Other fish products | 1.3 | 1.1 | 2.3 | 1.0 | 0.9 | 1.3 |
|  | | Shellfish | | | 1.7 | 0.1 | 0.5 | 0.3 | 3.2 | 3.1 |
|  | | | | | | | | | | |
| Fruit | | | | | 6.5 | 9.3 | 9.2 | 6.7 | 4.0 | 0.6 |
|  | | Fresh fruit | | | 4.2 | 6.8 | 4.9 | 4.0 | 2.7 | 0.2 |
|  | | Juice | | | 1.3 | 1.7 | 1.8 | 1.6 | 1.0 | 0.0 |
|  | | Other fruit | | | 1.0 | 0.8 | 2.5 | 1.1 | 0.3 | 0.3 |
|  | | | | | | | | | | |
| Grain products | | | | | 12.8 | 16.5 | 7.6 | 5.5 | 14.4 | 14.0 |
|  | | Bread | | | 8.5 | 13.4 | 6.0 | 3.3 | 7.7 | 9.7 |
|  | |  | | White bread | 2.8 | 4.5 | 2.0 | 1.3 | 2.5 | 2.9 |
|  | |  | | Wholegrain bread | 5.1 | 8.3 | 3.3 | 1.7 | 4.7 | 6.4 |
|  | |  | | Other bread | 0.5 | 0.6 | 0.7 | 0.2 | 0.5 | 0.5 |
|  | | Pastries | | | 2.8 | 1.2 | 0.9 | 0.9 | 4.9 | 2.4 |
|  | |  | | Buns | 2.1 | 0.8 | 0.7 | 0.7 | 3.8 | 1.8 |
|  | |  | | Biscuits | 0.1 | 0.2 | 0.0 | 0.0 | 0.1 | 0.0 |
|  | |  | | Other pastries | 0.6 | 0.2 | 0.2 | 0.1 | 1.0 | 0.6 |
|  | | Other grain products | | | 1.6 | 2.0 | 0.7 | 1.3 | 1.8 | 1.9 |
|  | |  | | Pizza | 0.8 | 1.2 | 0.3 | 0.9 | 0.7 | 1.1 |
|  | |  | | Cereal | 0.8 | 0.8 | 0.4 | 0.4 | 1.1 | 0.7 |
|  | | | | | | | | | | |
| Meat total | | | | | 10.2 | 3.6 | 3.7 | 4.9 | 16.5 | 23.8 |
|  | | Fresh meat | | | 3.7 | 0.7 | 1.4 | 2.4 | 5.8 | 12.3 |
|  | |  | | Poultry | 2.2 | 0.6 | 0.1 | 2.1 | 3.5 | 9.7 |
|  | |  | | Other fresh meat | 1.4 | 0.1 | 1.3 | 0.2 | 2.3 | 2.7 |
|  | | Meat products | | | 6.6 | 2.9 | 2.3 | 2.5 | 10.7 | 11.4 |
|  | |  | | Other meat products | 2.9 | 1.8 | 0.7 | 1.5 | 4.4 | 5.4 |
|  | |  | | Meat spread | 3.7 | 1.1 | 1.6 | 1.0 | 6.3 | 6.1 |
|  | | | | | | | | | | |
| Sweets and snacks | | | | | 0.5 | 0.7 | 0.1 | 0.2 | 0.7 | 0.0 |
|  | | Sugar and sweets | | | 1.2 | 1.0 | 2.5 | 1.5 | 0.6 | 1.0 |
|  | |  | | Sugar | 0.0 | 0.0 | 0.0 | 0.0 | 0.0 | 0.0 |
|  | |  | | Sweet spread | 0.0 | 0.1 | 0.0 | 0.0 | 0.0 | 0.0 |
|  | |  | | Sweets | 1.2 | 0.9 | 2.5 | 1.5 | 0.6 | 1.0 |
|  | | Snacks | | | 0.5 | 0.7 | 0.1 | 0.2 | 0.7 | 0.0 |
|  | |  | | Chips | 0.1 | 0.1 | 0.0 | 0.0 | 0.1 | 0.0 |
|  | |  | | Nuts seeds | 0.4 | 0.5 | 0.1 | 0.2 | 0.5 | 0.0 |
|  | |  | | Other snacks | 0.1 | 0.1 | 0.0 | 0.0 | 0.1 | 0.0 |
|  | | | | | | | | | | |
| Vegetables | | | | | 17.2 | 30.2 | 7.8 | 35.6 | 14.2 | 0.2 |
|  | Potatoes | | | | 6.3 | 12.1 | 5.4 | 8.6 | 4.2 | 0.0 |
|  | Fresh vegetables | | | | 10.3 | 17.4 | 2.3 | 26.6 | 9.2 | 0.0 |
|  |  | | Root vegetables | | 2.0 | 5.4 | 0.8 | 5.2 | 0.3 | 0.0 |
|  |  | | Leafy vegetables | | 5.7 | 8.2 | 0.9 | 15.1 | 6.2 | 0.0 |
|  |  | | Other vegetables | | 2.6 | 3.8 | 0.6 | 6.3 | 2.7 | 0.0 |
|  | Canned vegetables | | | | 0.6 | 0.8 | 0.1 | 0.4 | 0.8 | 0.2 |
